# Four new species of *Trigonopterus* Fauvel from the island of New Britain (Coleoptera, Curculionidae)

**DOI:** 10.3897/zookeys.582.7709

**Published:** 2016-04-21

**Authors:** Matthew H. Van Dam, Raymond Laufa, Alexander Riedel

**Affiliations:** 1 SNSB-Zoological State Collection, Münchhausenstr. 21, D-81247 Munich, Germany; 2 School of Natural & Physical Sciences, The University of Papua New Guinea, PO Box 320, UNIVERSITY 134, National Capital District, Papua New Guinea; 3 State Museum of Natural History Karlsruhe, Erbprinzenstr. 13, D-76133 Karlsruhe, Germany

**Keywords:** Bismarck Archipelago, *cox1*, Cryptorhynchinae, DNA barcoding, endemism, hyperdiverse, morphology, New Guinea, Nakanai Range, weevils

## Abstract

The hyperdiverse genus *Trigonopterus* has its center of diversity in Melanesia, but only a single species is recorded from the Bismarck Archipelago to date. Here we describe four new species from the island of New Britain: *Trigonopterus chewbacca* **sp. n.**, *Trigonopterus obsidianus* **sp. n.**, *Trigonopterus puncticollis* **sp. n.** and *Trigonopterus silaliensis* **sp. n.** We provide cytochrome oxidase subunit I (cox1) sequences of the new species and a key to all five species known from the Bismarck Archipelago.

## Introduction

*Trigonopterus* Fauvel is a genus of flightless weevils of the subfamily Cryptorhynchinae (Alonso-Zarazaga and Lyal 1999). It is distributed in Southeast Asia (Riedel et al. 2014), Australia (Riedel and Tänzler 2016) and Melanesia, with its center of diversity in New Guinea (Riedel et al. 2010; Tänzler et al. 2012, Riedel et al. 2013b). Despite *Trigonopterus* being recorded from the remote islands of Fiji (Zimmerman 1938b), Samoa (Marshall 1931) and New Caledonia (Heller 1916), only one species has been described from the Bismarck Archipelago to date, i.e. *Trigonopterus pembertoni* (Zimmerman 1938a) from New Ireland. Here we describe four new species from the island of New Britain. Presumably, there are many additional new species to be found on this island. Unfortunately, large expanses of low-elevation forests in New Britain have been converted to oil-palm plantations, highlighting the significance of documenting the insect fauna before the remaining forests are gone.

## Materials and methods

This study is based on 18 specimens, the result of a ten-day expedition to the area east of Silali Village in the Nakanai Range of West New Britain during November of 2014 by the first two authors. Specimens were collected by beating foliage and by sifting of leaf litter with subsequent extraction of specimens using Winkler eclectors (Besuchet et al. 1987). Holotypes were selected from the ten sequenced specimens; their DNA was non-destructively extracted as described by Riedel et al. (2010). Preparation of genitalia follows the method described by Riedel et al. (2014). Illustrations of habitus and genitalia are of holotypes.

Type depositories are cited using the following codes:

ANIC
Australian National Insect Collection, Canberra, Australia 


SMNK
Staatliches Museum für Naturkunde, Karlsruhe, Germany 


ZSM
Zoologische Staatssammlung, München, Germany 


UPNG
University of Papua New Guinea, Entomology Collection 


DNA sequencing and sequence analysis follows the method described by Riedel et al. (2010) and Tänzler et al. (2012). In the diagnostic descriptions, only the major characters are given, as in the format outlined by Riedel et al. (2013a, b).

Specimens were studied under a Leica MZ16 dissecting microscope and a fluorescent desk lamp for illumination. Measurements were taken using an ocular grid. Body length was measured in dorsal aspect from the elytral apex to the front of the pronotum, and elytral width between the humeri at their greatest extent and across *both* elytra. Legs were described in an idealized, laterally extended position; there is a dorsal / ventral and an anterior / posterior surface. Habitus illustrations were prepared by photographing the specimens with a DFC450 camera with L.A.S. 4.6.0 software mounted on a Z6 APO (all from Leica Microsystems, Heerbrugg, Switzerland). Photographs of the genitalia were taken under an Axio Imager M2 microscope (Carl Zeiss Microscopy) equipped with 5X or 10X A-Plan lenses and with a JVC KY70 camera (JVC Professional Products); the resulting image stacks were combined using the Helicon Focus 6.2.2 software (Helicon Soft Ltd). For this purpose the genitalia were embedded in glycerol gelatin, as described by Riedel (2005). The genitalia were photographed with their longitudinal axis somewhat raised at the posterior end, to adequately illustrate the structures of the curved apex. All photographs were enhanced using Adobe Photoshop CS2. Sequence data were submitted to the GenBank, and the accession numbers are provided under each species e.g. as “(GenBank # KU888894)”.

## Taxonomy

### Trigonopterus

Taxon classificationAnimaliaColeopteraCurculionidae

Fauvel, 1862

#### Type species.

*Trigonopterus insignis* Fauvel, 1862, by monotypy.

#### Diagnosis.

Fully apterous genus of Cryptorhynchinae. Length 1.5–6.0 mm. Rostrum in repose not reaching middle of mesocoxal length. Scutellar shield completely covered by elytra. Mesothoracic receptacle deep, posteriorly closed. Metanepisternum completely absent. Metathoracic spiracles located externally on side of metaventrite. Elytra with 9 striae (sometimes superficially effaced). Tarsal claws minute. Body usually largely unclothed. For additional information, see van de Kamp et al. (2015) and http://species-id.net/wiki/Trigonopterus.

### Descriptions of the species

#### Trigonopterus chewbacca

Taxon classificationAnimaliaColeopteraCurculionidae

Van Dam & Riedel sp. n.

http://zoobank.org/1EA211AA-4D08-4B65-8C3B-9A305B8BD1C9

##### Diagnostic description.

**Figure 1.**
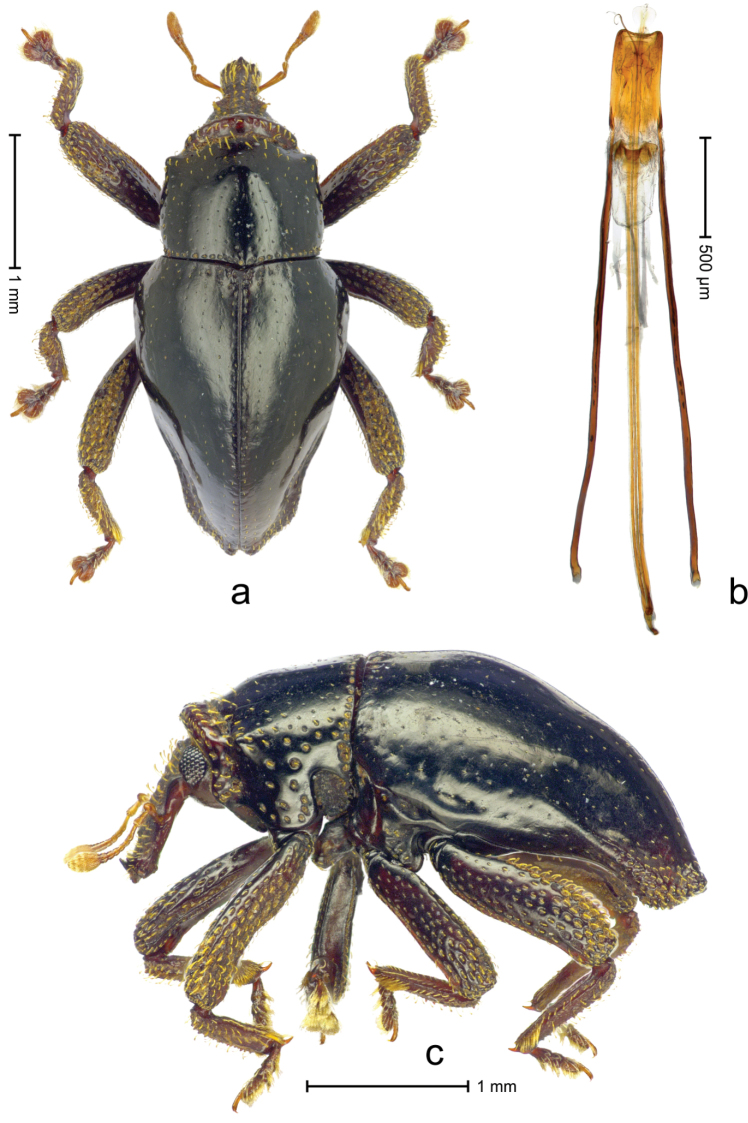
*Trigonopterus chewbacca* Van Dam & Riedel, sp. n., holotype; **a** Dorsal habitus **b** Lateral habitus **c** Penis.

Holotype, male (Fig. 1a–c). Length 3.34 mm. Color black; legs and antenna ferruginous. Body subrhomboid; in dorsal aspect with marked constriction between pronotum and elytron; in profile dorsally convex. Rostrum dorsally with rows of erect, clavate scales; with broad median costa bearing three fine ridges; with pair of sublateral furrows; epistome subglabrous, with sparse long setae, posteriorly forming transverse ridge bearing denticles; median denticle largest. Forehead in middle with denticle; laterally with row of long, erect, clavate scales bordering eye. Pronotum with subapical constriction; anteriorly with coarse punctures and sparse clavate scales, laterally angularly projecting; disk subglabrous, with sparse small punctures; basal margin bordered by row of coarse punctures; laterally subglabrous with sparse coarse punctures, posteriorly with large fovea. Elytra subglabrous, striae weakly marked by rows of minute punctures; intervals flat; laterally few punctures deeply impressed; apex subangulate, medially with suture incised, in profile curved ventrad, slightly beak-shaped, punctate-granulate, with sparse recumbent scales. Femora edentate, coarsely punctate, with sparse suberect scales. Metafemur with dorsoposterior edge markedly denticulate; subapically without stridulatory patch. Tibiae dorsally denticulate, with row of erect scales. Abdominal ventrites 1–2 forming common, subglabrous cavity; lateral rim with sparse scales; ventrite 5 with broad, subglabrous impression, subapically coarsely punctate. Penis (Fig. 1b) with apodemes and transfer apparatus in repose reaching into prothorax, > 4 × longer than body of penis; sides of body parallel, apex subangulate, medially with sparse setae; transfer apparatus flagelliform, enveloped by sclerotized sheath; endophallus at base of body with funnel-shaped sclerite; ductus ejaculatorius basally sclerotized, apical portion broken and missing. **Intraspecific variation**. Length 2.78–3.13 mm. Female rostrum with median ridge only basally; in apical third punctate, epistome without distinct transverse ridge.

##### Material examined.

Holotype (ANIC): ARC4224 (GenBank # KU888903), Papua New Guinea: West New Britain Prov.: E of Silali Village, Nakanai Range, S05°31.233', E151°03.343', 800 m, from leaf litter, 21-22-XI-2014. Paratypes (SMNK, UPNG): 2 exx, ARC4222 (GenBank # KU888901), ARC4223 (GenBank # KU888902), same data as holotype.

##### Etymology.

This epithet is a noun in apposition and based on the likeable fictional character Chewbacca in George Lucas’ Star Wars movies, portrayed primarily by Peter Mayhew. This species has dense scales on the head and the legs, which reminds the authors of Chewbacca’s dense fur.

##### Notes.

Presumably, the species belongs in the *Trigonopterus basalis*-group of Riedel et al. (2013b).

#### Trigonopterus obsidianus

Taxon classificationAnimaliaColeopteraCurculionidae

Van Dam & Riedel sp. n.

http://zoobank.org/8AE75F70-A3F7-48EC-93C7-C49B2984C215

##### Diagnostic description.

**Figure 2.**
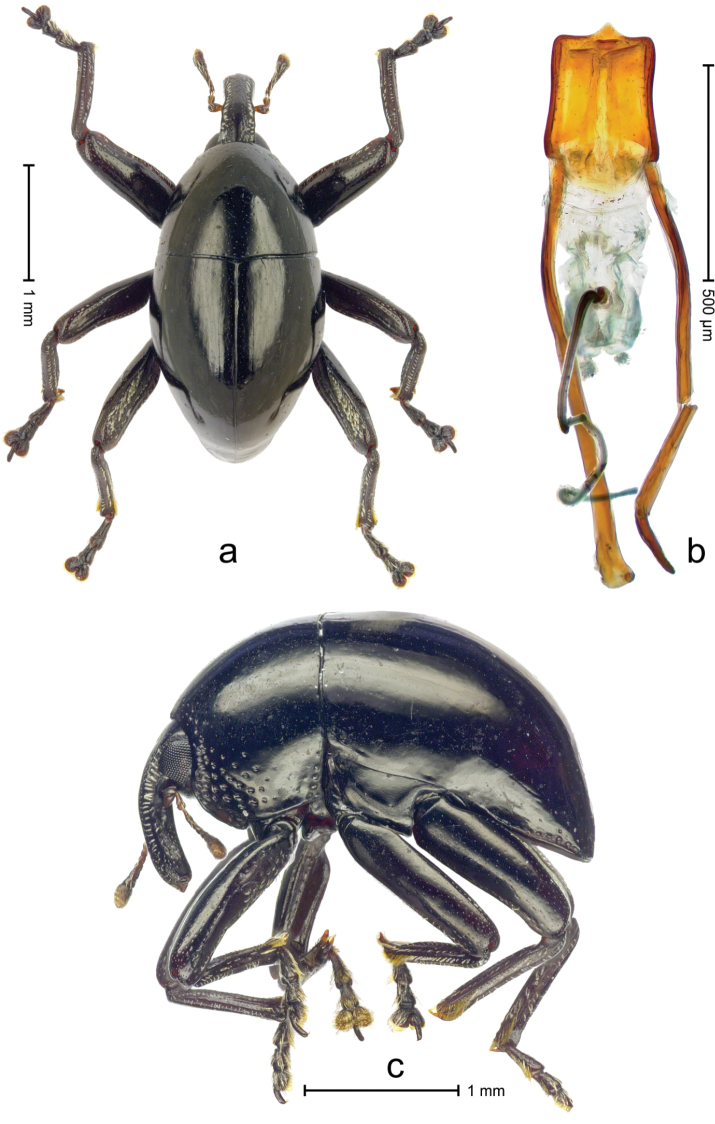
*Trigonopterus obsidianus* Van Dam & Riedel, sp. n., holotype; **a** Dorsal habitus **b** Lateral habitus **c** Penis.

Holotype (Fig. 2a–c). Length 2.85 mm. Color black. Body subovate, almost without constriction between pronotum and elytron; in profile evenly convex. Rostrum dorsally sparsely punctate, with pair of sublateral furrows containing rows of scales directed mesad; in front of antennal insertion with shallow lateral constriction. Eye with dorsal margin weakly carinate, bordered by furrow. Forehead with sparse minute punctures. Pronotum with disk subglabrous, with minute punctures; sides above coxa with scattered, coarse punctures; base medially weakly extended towards elytral suture. Elytra subglabrous, including near base and humeri; striae hardly visible. Legs. Femora subglabrous, with minute punctures. Metafemur dorsally with elongate patch of dense silvery scales; posterior surface with pair of longitudinal furrows containing rows of indistinct scales parallel to ventral and dorsal edge; dorsoposterior edge distinct. Mesotibia apically with uncus and separate, much larger premucro. Metatibia in subapical third ventrally with weak swellings, but not denticulate; apically with uncus but no premucro. Abdominal ventrites 1 and 2 medially forming common cavity; ventrite 2 laterally swollen and with suberect scales; posterior edge projecting; ventrite 5 flat, subglabrous, dull, with sparse minute punctures. Penis (Fig. 2b) with sides of body subparallel, weakly concave; apex with median triangular extension, symmetrical; endophallus with short, spiniform transfer apparatus directed ventrad in repose, without distinct sclerites; ductus ejaculatorius without bulbus. **Intraspecific variation**. Length 2.58–2.85 mm. Female mesotibia apically with large uncus and much smaller premucro. Female abdominal ventrites 1 and 2 medially flat.

##### Material examined.

Holotype (ANIC): ARC4216 (GenBank # KU888896), Papua New Guinea: West New Britain Prov.: E of Silali Village, Nakanai Range, S05°31.233', E151°03.343', 800 m, from foliage, 21-22-XI-2014. Paratypes (SMNK, UPNG): 2 exx, ARC4215 (GenBank # KU888895), ARC4217 (GenBank # KU888897), same data as holotype.

##### Etymology.

This epithet is based on the Latin adjective *obsidianus* and refers to the color of the polished mineral obsidian, which resembles the pronotum and elytra of this species.

##### Notes.

This species belongs to the *Trigonopterus politus*-group of Riedel et al. (2013b) and is similar to *Trigonopterus politus* (Faust) of the Papuan Peninsula. It can be distinguished by the symmetrical apex of the penis and the less distinct denticles of the male metatibia.

#### Trigonopterus puncticollis

Taxon classificationAnimaliaColeopteraCurculionidae

Van Dam & Riedel sp. n.

http://zoobank.org/69951605-F4A2-4EE1-94B0-6A771B7236CA

##### Diagnostic description.

**Figure 3.**
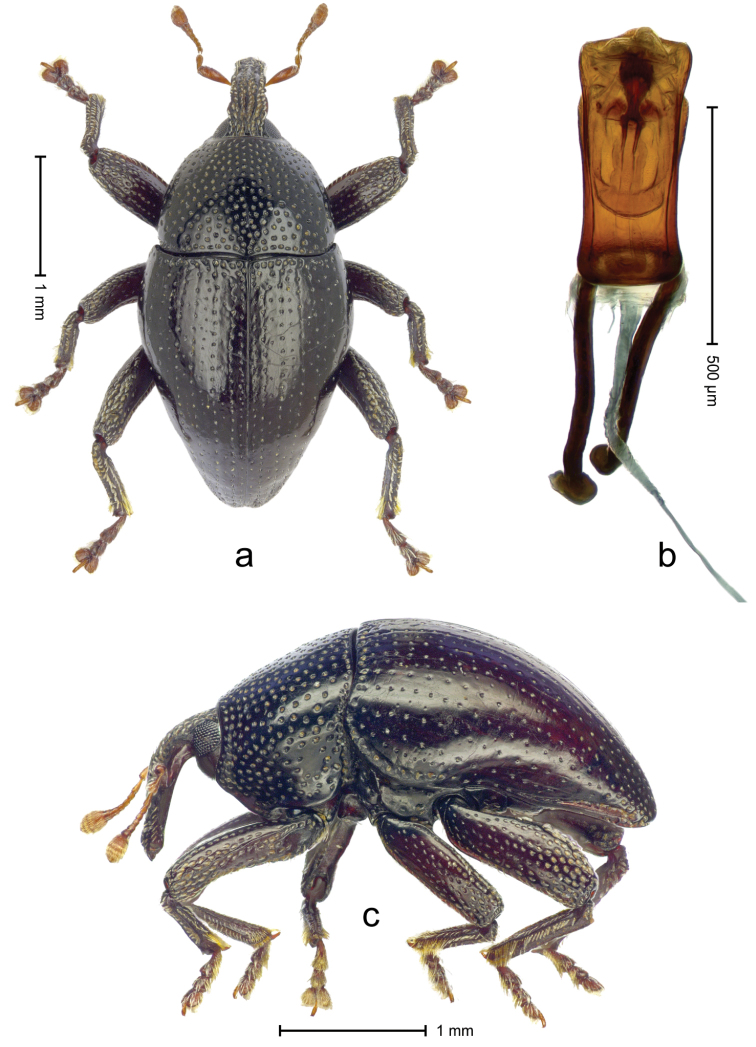
*Trigonopterus puncticollis* Van Dam & Riedel, sp. n., holotype; **a** Dorsal habitus **b** Lateral habitus **c** Penis.

**Holotype**, male (Fig. 3a–c). Length 3.06 mm. Color black, antenna and tarsi ferruginous. Body subovate; with weak constriction between pronotum and elytra; in profile evenly convex. Rostrum in basal half with median and pair of submedian carinae, intervening furrows with rows of yellowish-transparent scales; apical half of rostrum coarsely punctate-rugose, with sparse suberect setae. Forehead coarsely punctate-rugose, punctures with subrecumbent scales pointing backwards. Pronotum coarsely punctate; distance between punctures subequal to their diameter; each puncture containing one inconspicuous seta. Elytra finely punctate, along basal margin with transverse row of deeper and denser punctures; striae impressed as fine lines; marked by rows of small punctures; intervals flat, subglabrous, with few minute punctures; lateral stria behind humerus with dense row of deep punctures. Femora edentate; subapically coarsely punctate, with recumbent scales. Anteroventral ridge of profemur in apical third shortened, forming blunt angulation. Metafemur dorsoposteriorly denticulate; subapically without stridulatory patch. Protibia widening towards apex. Mesotibia in apical half with anterior face covered by fringe of long subrecumbent setae. Meso- and metatibia subbasally with dorsal angulation; metatibia with supra-uncal denticle. Abdominal ventrites 1–2 concave with coarse punctures bearing silvery plumose scales; ventrite 5 flat, densely punctate and covered with silvery scales. Penis (Fig. 3b) widening apicad, in apical third with constriction and oblique lateral furrow; apex subangulate, subglabrous; transfer apparatus symmetrical, contained in apical half of body; ductus ejaculatorius without bulbus. **Intraspecific variation**. Length 2.58–2.85 mm. Female mesotibia apically with large uncus and much smaller premucro. Female abdominal ventrites 1 and 2 medially flat.

##### Material examined.

Holotype (ANIC): ARC4220 (GenBank # KU888900), PAPUA NEW GUINEA, West New Britain Prov.: E of Silali Village, Nakanai Range, S 05°30.651', E 151°02.895', 700 m, from foliage, 21-XI-2014. Paratypes (SMNK, ZSM, UPNG): 9 exx, ARC4218 (EMBL # KU888898), ARC4219 (GenBank # KU888899), ARC4221 (EMBL # XXX), same data as holotype.

##### Etymology.

This epithet is a Latin adjective based on a combination of the Latin nouns *punctum* (small hole, dot) and *collum* (neck) and refers to the markedly punctate pronotum.

##### Notes.

This species may belong to the *Trigonopterus oblongus*-group of Riedel et al. (2013b) in a wide sense.

#### Trigonopterus silaliensis

Taxon classificationAnimaliaColeopteraCurculionidae

Van Dam & Riedel sp. n.

http://zoobank.org/C6C79A9A-0A51-4252-9603-AF408E46DF26

##### Diagnostic description.

**Figure 4.**
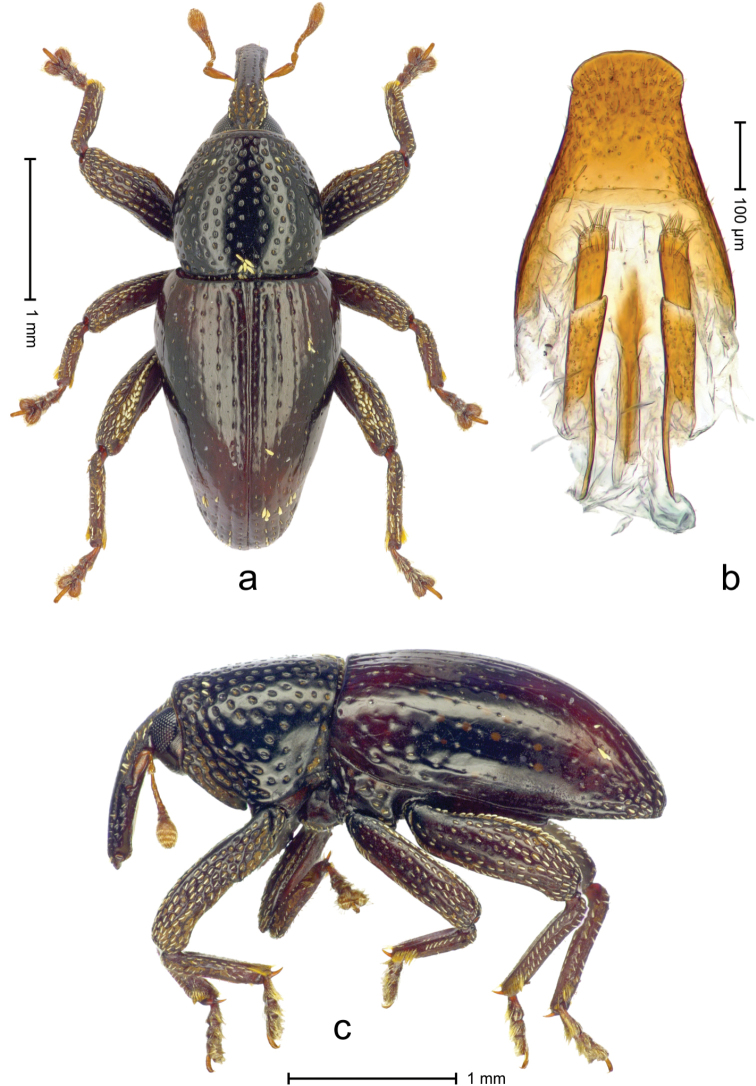
*Trigonopterus silaliensis* Van Dam & Riedel, sp. n., holotype; **a** Dorsal habitus **b** Lateral habitus **c** female genitalia.

**Holotype**, female (Fig. 4a–c). Length 3.02 mm. Color black; elytra with humeri and apical third dark ferruginous; antenna paler ferruginous. Body elongate; in dorsal aspect with marked constriction between pronotum and elytra; in profile dorsally flat. Rostrum basally with median and pair of submedian carinae; in apical half relatively smooth, with submedian row of punctures. Forehead coarsely punctate-rugose, with cream-colored, narrow scales directed backwards. Pronotum with sides convex, without subapical constriction; disk dorsally flat, punctate, at middle subglabrous, punctures sparse; anterolaterally punctures coarse, partly confluent; at middle of basal margin with small patch of dense, cream-colored recumbent scales. Elytra with striae distinct, dorsally with rows of small punctures, laterally with deep punctures; intervals flat, subglabrous; with small, scattered patches of cream-colored, recumbent scales. Legs. Femora edentate; coarsely punctate, with white, recumbent piliform scales. Profemur in basal third posteriorly with callus. Metafemur dorsally with white scales wider and more densely; subapically with stridulatory patch. Abdominal ventrites flat, with fine punctures bearing piliform scales. Genitalia. Fig. 4b.

##### Material examined.

Holotype (ANIC): ARC4214 (GenBank # KU888894), PAPUA NEW GUINEA, West New Britain Prov.: E of Silali Village, Nakanai Range, S05°31.233', E151°03.343', 800 m, from foliage, 21-22-XI-2014.

##### Etymology.

This epithet is a Latin adjective based on the name of the village near to which the holotype was collected.

##### Notes.

This species belongs to the *Trigonopterus honestus*-group of Riedel et al. (2013b) and superficially resembles *Trigonopterus paucisquamosus* (Heller) from the Philippines, which differs by the position of scale patches and a denser punctation.

### Key to the known *Trigonopterus* species of the Bismarck Archipelago of Papua New Guinea

**Table:** 

1	Species found on foliage. Pronotum subapically rounded, without distinct constriction	**2**
–	Species found in the litter layer. Pronotum subapically with distinct constriction and pair of angular projections (Fig. 1a–c)	***Trigonopterus chewbacca* Van Dam & Riedel, sp. n.**
2(1)	Body larger, pronotum plus elytron ca. 5.63 mm. Elytron black, nude except subapically with elongate patch of white scales. Fig. 5.	***Trigonopterus pembertoni* (Zimmerman)**
–	Body smaller, pronotum plus elytron ca. 3.02–3.06 mm. Elytron without subapical patch of white scales.	**3**
3(2)	Pronotum smooth, almost impunctate. Male mesotibia subapically with premucro larger than uncus	***Trigonopterus obsidianus* Van Dam & Riedel, sp. n.**
–	Pronotum densely punctate. Male mesotibia subapically with uncus; premucro minute or absent	**4**
4(3)	Body elongate. Metafemur subapically with stridulatory patch	***Trigonopterus silaliensis* Van Dam & Riedel, sp. n.**
–	Body subovate. Metafemur without stridulatory patch	***Trigonopterus puncticollis* Van Dam & Riedel, sp. n.**

**Figure 5.**
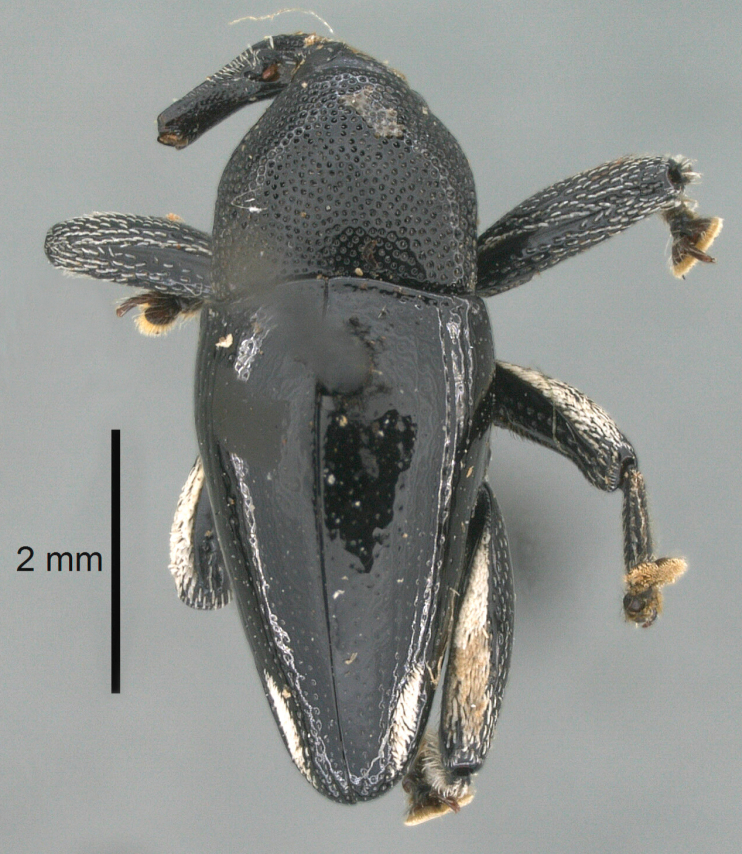
*Trigonopterus pembertoni* (Zimmerman), holotype. Dorsal habitus. Photo courtesy B.P. Bishop Museum.

## Discussion

The absence of a record of a weevil genus from a Melanesian island is often difficult to interpret, i.e. it is usually unclear whether this is based on a true absence or on a lack of records. Prior to this study, *Trigonopterus* was unknown from New Britain. The four species described here represent four different clades of *Trigonopterus*, indicating that the oceanic island of New Britain has been colonized at least four times, and *Trigonopterus pembertoni*, which occurs on neighboring New Ireland and represents the *Trigonopterus oblongus*-group, brings the number of colonization events of *Trigonopterus* in the Bismarck Archipelago to five. Given the size, mountainous topography and tropical vegetation of New Britain, it is likely that *Trigonopterus* has undergone some local speciation on the island, but this possibility requires further investigation.

Despite many days of searching for *Trigonopterus* in primary forest on New Britain, the weevils were quite scarce in comparison with similar localities on the New-Guinean mainland. This scarcity may be due to the local conditions or seasonal effects, as orographic precipitation formed early in the day and continued into the evening during our stay. The specimens’ habitat consisted of primary forest growing on a limestone karst.

## Supplementary Material

XML Treatment for Trigonopterus

XML Treatment for Trigonopterus chewbacca

XML Treatment for Trigonopterus obsidianus

XML Treatment for Trigonopterus puncticollis

XML Treatment for Trigonopterus silaliensis
